# Identification of potential crucial genes and therapeutic targets for epilepsy

**DOI:** 10.1186/s40001-024-01643-8

**Published:** 2024-01-11

**Authors:** Shitao Wang, Zhenrong Xie, Tian Jun, Xuelu Ma, Mengen Zhang, Feng Rao, Hui Xu, Jinghong Lu, Xiangqian Ding, Zongyou Li

**Affiliations:** 1 Department of Neurology, Affiliated Fuyang People’s Hospital of Anhui Medical University, Fuyang, 236000 Anhui China; 2 The Medical Biobank, First Affiliated Hospital of Kunming Medical University, Kunming, 650032 Yunnan China; 3 Department of Neurosurgery, Qilu Hospital of Shandong University, Jinan, 250012 Shandong China

**Keywords:** Gene, Epilepsy, *PTPRO*, *GADD45A*, Target

## Abstract

**Background:**

Epilepsy, a central neurological disorder, has a complex genetic architecture. There is some evidence suggesting that genetic factors play a role in both the occurrence of epilepsy and its treatment. However, the genetic determinants of epilepsy are largely unknown. This study aimed to identify potential therapeutic targets for epilepsy.

**Methods:**

Differentially expressed genes (DEGs) were extracted from the expression profiles of GSE44031 and GSE1834. Gene co-expression analysis was used to confirm the regulatory relationship between newly discovered epilepsy candidate genes and known epilepsy genes. Expression quantitative trait loci analysis was conducted to determine if epilepsy risk single-nucleotide polymorphisms regulate DEGs’ expression in human brain tissue. Finally, protein–protein interaction analysis and drug–gene interaction analysis were performed to assess the role of DEGs in epilepsy treatment.

**Results:**

The study found that the protein tyrosine phosphatase receptor-type O gene (*PTPRO*) and the growth arrest and DNA damage inducible alpha gene (*GADD45A*) were significantly upregulated in epileptic rats compared to controls in both datasets. Gene co-expression analysis revealed that *PTPRO* was co-expressed with *RBP4*, *NDN*, *PAK3*, *FOXG1*, *IDS*, and *IDS*, and *GADD45A* was co-expressed with *LRRK2* in human brain tissue. Expression quantitative trait loci analysis suggested that epilepsy risk single-nucleotide polymorphisms could be responsible for the altered *PTPRO* and *GADD45A* expression in human brain tissue. Moreover, the protein encoded by *GADD45A* had a direct interaction with approved antiepileptic drug targets, and *GADD45A* interacts with genistein and cisplatin.

**Conclusions:**

The results of this study highlight *PTPRO* and *GADD45A* as potential genes for the diagnosis and treatment of epilepsy.

**Supplementary Information:**

The online version contains supplementary material available at 10.1186/s40001-024-01643-8.

## Background

Epilepsy, a central neurological disorder characterized by recurrent, spontaneous seizures, affects over 68 million people globally [1]. The primary causes of epilepsy are abnormal discharges in the hippocampus or cerebral cortex [2, 3]. While approximately 70% of seizures can be effectively managed with approved antiepileptic drugs [4], the remaining 30% are resistant to pharmacotherapy, resulting in significant psychological and physical burdens for patients [5].

The genetic determinants of epilepsy are largely unknown. There is evidence suggesting that genetic factors contribute to both generalized and focal epilepsies [6]. Mutations in certain genes have been associated with epilepsy [6, 7], but the role of common polymorphisms in epilepsy is still unclear [8, 9]. Several recent studies have identified new epilepsy loci [10–15], and further expanded analysis has revealed additional new loci for epilepsy [16]. Moreover, gene therapy has been reported as a potential treatment for refractory focal epilepsy [17].

Bioinformatics-based studies using microarray analysis are crucial for identifying disease-related gene expression patterns [18–21]. In this study, we analyzed gene expression between epileptic rat models and healthy samples to investigate their potential association with epilepsy. We further conducted integrative analyses with gene co-expression, expression quantitative trait loci (eQTL), protein–protein interaction (PPI) networks, and drug–gene interaction to clarify the potential role of newly discovered epilepsy-associated genes in the diagnosis and treatment of epilepsy.

## Materials and methods

### Gene Expression Omnibus datasets

In this study, we utilized two independent datasets from the Gene Expression Omnibus (GEO). These datasets, GSE44031 [22] and GSE1834 [23], were analyzed using the GPL9207 platform (Duke Operon Rat 27 k V3.0 printed oligonucleotide array) and the GPL85 platform ([RG_U34A] Affymetrix Rat Genome U34 Array), respectively. For detailed information on these datasets, please refer to the GEO database (http://www.ncbi.nlm.nih.gov/geo).

### Differentially expressed genes (DEGs) analysis

Differentially expressed genes (DEGs) were identified using R software. The gene expression levels were extracted from the GSE44031 dataset, which includes microarray data from eight hippocampal tissue samples from epileptic rats induced by kainic acid injection, and four samples from rats injected with phosphate buffer saline. These differentially expressed genes were further validated in the GSE1834 dataset, which contains data from 15 hippocampal tissue samples from epileptic rats induced by kainic acid injection, and 15 samples from rats injected with phosphate buffer saline. A statistical significance threshold was set at an adjusted *p* value ≤ 0.05 and |logFold change|≥ 1. The overlapping DEGs were selected for further analysis using a Venn map, which was created using the Venn online tool (http://bioinformatics.psb.ugent.be/webtools/Venn/).

### Gene co-expression analysis

Gene co-expression analysis is a method used to discover the regulatory relationships between genes and subsequently identify potential disease candidate genes. Given that gene expression is tissue-specific, we investigated the co-expression relationships between DEGs and known epilepsy genes in human brain tissues. The gene co-expression database for brain tissue serves as an effective tool for analyzing the co-expression relationships of genes associated with brain diseases [24].

### eQTL analysis

To determine the impact of epilepsy risk single-nucleotide polymorphisms (SNPs) on the expression of DEGs in human brain tissue, we performed eQTL analysis. This analysis was conducted using BRAINEAC (http://www.Braineac.org/) [25], a tool designed to investigate the association between SNPs and the expression of their target genes in human brain tissue.

### Evaluation of DEGs in epilepsy treatment

To identify new antiepileptic drug targets and facilitate the translation of these findings into clinical therapy, we first identified approved antiepileptic drug therapeutic targets using DrugBank5.0 and the Therapeutic Target Database 2020 [26, 27]. We then conducted PPI analysis to explore the relationship between newly discovered epilepsy-related genes and approved antiepileptic drug target genes. This PPI analysis was performed using the STRING database (https://string-db.org/cgi/input.pl) [28], and a PPI network was constructed using Cytoscape software [29]. The findings from the PPI analysis were further validated by a drug–gene interaction analysis using the Drug–Gene Interaction Database [30].

## Result

### Analysis of DEGs in epilepsy rat models and controls

**Fig. 1:**
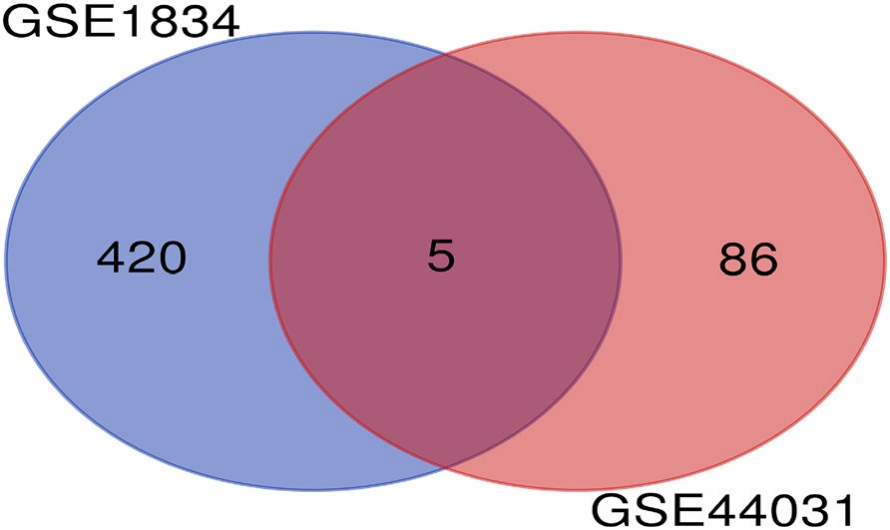
DEGs were identified from GSE44031 and GSE1834 gene expression profiling datasets based on adjusted p value < 0.05 and |logfold change**|**≥ 1. The two datasets share 5 overlapping DEGs

**Fig. 2:**
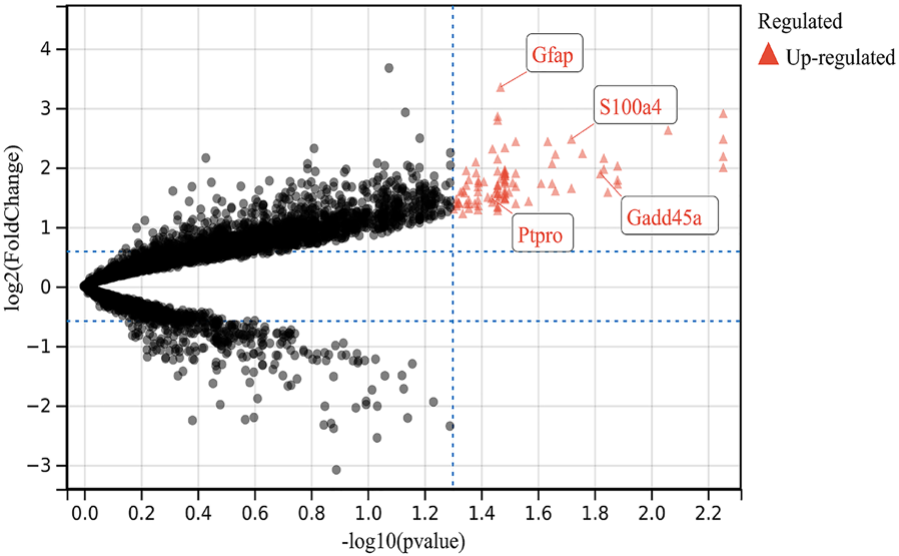
The volcano plot illustrates DEGs. The volcano plot illustrates DEGs between epilepsy and control after analysis of the GSE44031 gene expression profiling datasets

Based on the selection criteria for DEGs outlined in the “Methods” section, 91 DEGs were selected from the GSE44031 dataset (see Additional file 1: Table S1), and 425 DEGs were selected from the GSE1834 dataset (see Additional file 2: Table S2). As depicted in Fig. 1, five genes overlapped between the two datasets (see Additional file 3: Table S3). Specifically, among these five DEGs, the expression regulation of one gene was inconsistent across the two datasets, leading to its removal. The expression of the remaining four genes was upregulated in both datasets (Fig. 2), with two of them (*GFAP* and *S100A4*) previously reported to be associated with epilepsy [31, 32]. Consequently, we selected two newly discovered candidate genes, the protein tyrosine phosphatase receptor-type O gene (*PTPRO*) and the growth arrest and DNA damage inducible alpha gene (*GADD45A*), for further analysis. The Volcano map of DEGs was created using the online volcano plotting tool (http://sangerbox.com/AllTools?tool_id=9699135).

### Gene co-expression analysis

**Fig. 3:**
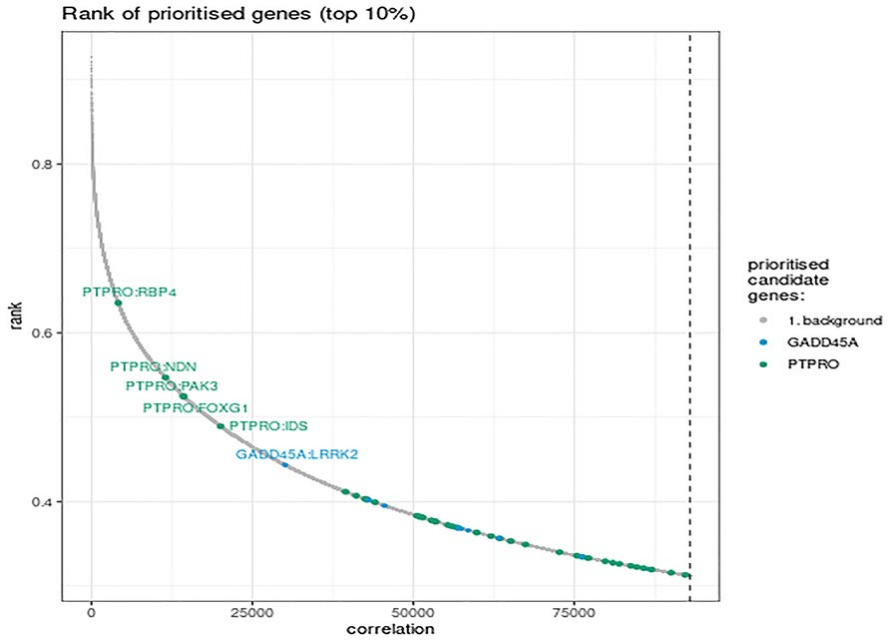
Analysis of gene co-expression in human brain. *PTPRO* was co-expressed with *RBP4*, *NDN*, *PAK3*, *FOXG1*, *IDS*, and *IDS*, and *GADD45A* was co-expressed with *LRRK2* in human brain tissue

As genetic research advances, an increasing number of epilepsy-related genes have been identified [33]. In this study, we utilized these reported epilepsy-related genes along with the newly discovered genes for gene co-expression analysis. The analysis revealed that *PTPRO* was co-expressed with *RBP4*, *NDN*, *PAK3*, *FOXG1*, and *IDS* in human brain tissue. Similarly, *GADD45A* was found to be co-expressed with *LRRK2* in human brain tissue (Fig. 3).

### eQTL analysis

In this study, we focused on SNPs that have been previously reported to be associated with epilepsy for eQTL analysis. Specifically, we explored whether the SNPs rs4794333, rs68082256, rs11943905, rs12185644, rs6432877, rs2212656, rs1402398, rs11890028, rs887696, rs1044352, rs13200150, and rs4671319, which have been associated with epilepsy in a multicenter study [16], regulate the expression levels of the newly discovered genes in human brain tissue. Interestingly, our findings suggest that these SNPs do indeed regulate the expression levels of *PTPRO* and *GADD45A* in human brain tissue (Table 1).

**Table 1:** Epilepsy risk SNPs regulate *GADD45A* and *PTPRO* expression in human brain tissue

SNP	Target gene	eQTL-p	Human brain tissue
Rs4794333	*GADD45A*	0.0037	Thalamus
Rs68082256	*GADD45A*	0.022	Medulla
Rs11943905	*GADD45A*	0.045	Temporal cortex
Rs12185644	*GADD45A*	0.041	Temporal cortex
Rs6432877	*PTPRO*	0.035	Occipital cortex
Rs2212656	*PTPRO*	0.031	Occipital cortex
Rs1402398	*PTPRO*	0.042	Putamen
Rs11890028	*PTPRO*	0.016	Frontal cortex
Rs887696	*PTPRO*	0.036	Occipital cortex
Rs1044352	*PTPRO*	0.0052	Occipital cortex
Rs13200150	*PTPRO*	0.037	Temporal cortex
Rs4671319	*PTPRO*	0.036	Temporal cortex

*SNP* single-nucleotide polymorphism, *eQTL* expression quantitative trait loci

### Evaluation of *PTPRO* and *GADD45A* in epilepsy treatment

**Fig. 4:**
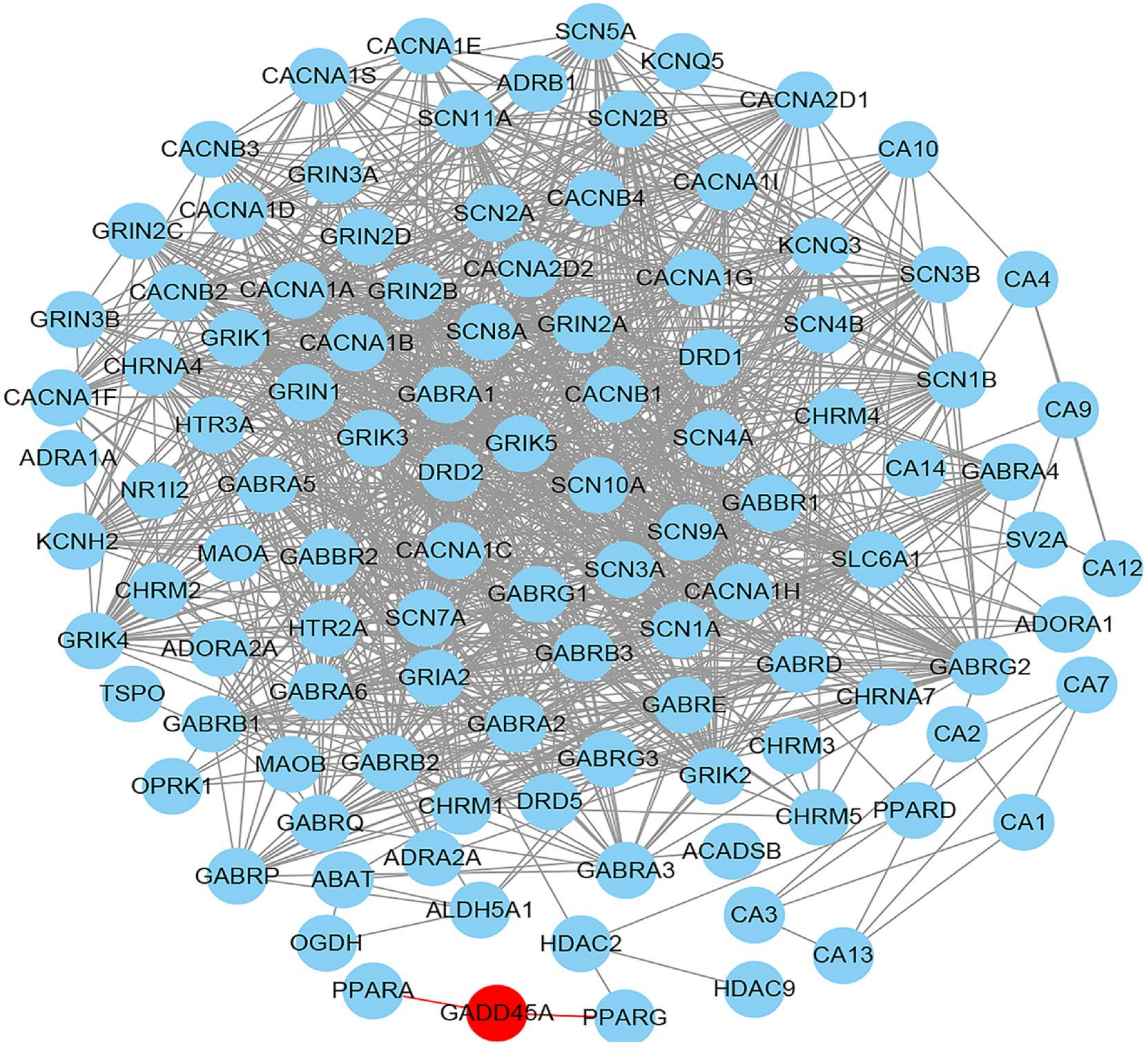
The PPI network of *PTPRO* and *GADD45A* and the genes whose encoded protein targeted by antiepileptic drugs. The proteins connected by the red line mean that they have direct interactions

To explore the potential role of *PTPRO* and *GADD45A* in epilepsy treatment, we first conducted an interaction analysis between the proteins encoded by *PTPRO* and *GADD45A* and the targets of approved antiepileptic drugs (Table 2). We found that the protein encoded by *GADD45A* had direct interactions with the targets of approved antiepileptic drugs, including PPARA and PPARG (Fig. 4). Further drug–gene interaction analysis revealed that *GADD45A* interacts with genistein and cisplatin.

**Table 2:** 115 genes encoding proteins targeted by approved antiepileptic drugs

Epilepsy drugs	Gene
Phenobarbital, Primidone, Phenytoin, Carbamazepine, Valproate, Clonazepam, Clobazam, Gabapentin, Lamotrigine, Topiramate, Oxcarbazepine, Tiagabine, Levetiracetam, Zonisamide, Felbamate, Pregabalin, Vigabatrin	*NR1I2, GRIA2, GABRA1, CHRNA4, CHRNA7, GRIK2, GRIN1, GRIN2A, GRIN2B, GRIN2C, GRIN2D, GRIN3A, GRIN3B, GABRA3, GABRA6, GABRA5, GABRB2, GABRB3, GABRD, GABRE, GABRG1, GABRG2, GABRG3, GABRP, GABRQ, SCN5A, SCN3A, CACNA1C, CACNA1D, CACNA1F, CACNA1S, CACNB1, CACNB2, CACNB3, CACNB4, CACNA1A, SCN8A, KCNH2, SCN1A, SCN1B, SCN2A, SCN4A, SCN7A, SCN9A, SCN10A, SCN11A, ALDH5A1, HDAC2, PPARA, PPARD, OGDH, SCN2B, SCN3B, SCN4B, PPARG, HDAC9, ACADSB, TSPO, CACNA2D1, KCNQ5, ADORA1, CACNA1B, CACNA2D2, KCNQ3, HTR3A, CACNA1E, ADRA2A, HRH1, OPRK1, ADORA2A, ADRA1A, DRD2, GABRA4, ADRB1, DRD1, DRD5, GABRA2, GABRB1, CHRM1, CHRM2, CHRM3,CHRM4, CHRM5, HTR2A, CA2, GRIK1, GRIK3, GRIK4, GRIK5, CA3, CA4, SLC6A1, SV2A, CA5B, CA10, CA11, CA12, CA13, MAOB, CA7, CA9, CACNA1H, CA1, CACNA1G, CA5A, CA6, CA8, CA14, CACNA1I, MAOA, ABAT, GABBR1, GSK3A, GABBR2, SLC5A6*

## Discussion

In this comprehensive analysis of epilepsy, we identified two new potential genes, *PTPRO* and *GADD45A*, and highlighted their crucial roles in epilepsy. Our results suggest that *GADD45A* might serve as a potential therapeutic target for epilepsy. The Consortium on Complex Epilepsies has made significant strides in identifying several loci with genome-wide significance for epilepsy [15, 16]. Research aimed at understanding the role of genes in epilepsy will contribute to a deeper understanding of the genetic architecture of epilepsy. To the best of our knowledge, the association of *PTPRO* and *GADD45A* with epilepsy has not been evaluated previously.

In this study, we initially identified five DEGs that overlapped in the GSE44031 and GSE1834 gene expression profiles. Further analysis revealed that two of these DEGs, *PTPRO* and *GADD45A*, had not been previously reported to be associated with epilepsy. Gene co-expression analysis uncovered specific regulatory relationships between these two newly discovered epilepsy-associated genes and known epilepsy genes (Fig. 3). These findings suggest that these potential genes are functionally associated with the reported epilepsy genes, further underscoring the role of these newly discovered genes in epilepsy. This could facilitate the diagnosis and treatment of epilepsy and may provide a new direction in the understanding of the disease.

SNPs located in noncoding regions can influence disease risk by regulating the expression of their target genes [34, 35]. Our eQTL analysis revealed that several noncoding SNPs associated with epilepsy risk alter the expression of *PTPRO* and *GADD45A* in brain tissue (Table 1). Moreover, these SNPs have been reported to be associated with epilepsy [16]. Therefore, we hypothesize that these SNPs may contribute to epilepsy risk by regulating *PTPRO* and *GADD45A* expression in human brain tissue. These findings further elucidate the mechanisms of *PTPRO* and *GADD45A* in the pathogenesis of epilepsy.

*PTPRO*, located on chromosome 12, encodes the receptor-type tyrosine-protein phosphatase O. It is highly expressed in the brain and promotes the formation of excitatory synapses [36]. *PTPRO* also plays a role in regulating the development and function of the sensory nervous system [37]. A genome-wide study has shown that *PTPRO* is associated with learning and memory [38]. On the other hand, *GADD45A* has been found to influence cortical evolution and diversity depending upon its expression levels [39, 40]. Therefore, both *PTPRO* and *GADD45A* may play significant roles in the pathogenesis of brain diseases.

PPI networks play a crucial role in drug target discovery and present drug discovery process [41]. Our PPI analysis revealed that the protein encoded by *GADD45A* directly interacts with the targets of approved antiepileptic drugs, PPARA and PPARG (Fig. 4). By integrating data from DrugBank5.0 and the Therapeutic Target Database 2020, we found that PPARA and PPARG are targets of Valproate. Furthermore, we discovered that *GADD45A* interacts with genistein and cisplatin. Genistein has been implicated in antiepileptic effects [42, 43], while cisplatin has been reported to induce seizures [44, 45]. Therefore, the results of the PPI networks and drug–gene interaction further underscore the significant role of *GADD45A* in epilepsy therapy. While recent studies have discovered new epilepsy-related genes [46, 47], our study not only identified new epilepsy-related genes but also explored the potential mechanisms of these new genes in the pathogenesis and treatment of epilepsy, which could be more conducive to the transformation of clinical application.

While our study integrated data from gene expression, gene co-expression, eQTL, PPI network, and drug–gene interaction to uncover the role of *PTPRO* and *GADD45A* in epilepsy diagnosis and therapy, there are still some limitations. First, further validation of our findings in independent populations is necessary to strengthen our conclusions. Second, additional functional characterization would help to better understand the mechanisms of *PTPRO* and *GADD45A* in the pathogenesis and treatment of epilepsy.

## Conclusion

In summary, our findings underscore the potential of *PTPRO* and *GADD45A* as promising targets for the diagnosis and treatment of epilepsy.

### Supplementary Information

**Additional file 1: Table S1.** 91 DEGs were identified in GSE44031 series.

**Additional file 2: Table S2.** 425 DEGs were identified in GSE1834 series.

**Additional file 3: Table S3.** 5 overlapping DEGs were identified in GSE1834 and GSE44031.

## Data Availability

The datasets generated during the current study are available in the GEO repository.

## Abbreviations

DEGs: Differentially expressed genes
eQTL: Expression quantitative trait loci
PPI: Protein–protein interaction
SNPs: Single-nucleotide polymorphisms
*GFAP*: Glial Fibrillary Acidic Protein
*S100A4*: S100 Calcium-Binding Protein A4
*PTPRO*: Protein Tyrosine Phosphatase Receptor Type O
*GADD45A*: Growth Arrest and DNA Damage Inducible Alpha
