# Supplementary material for: Identification of potential crucial genes and therapeutic targets for epilepsy
Source: Eur J Med Res. 2024 Jan 11;29:43. doi: 10.1186/s40001-024-01643-8 (PMC10782668; doi:10.1186/s40001-024-01643-8)
Supplement: Supplementary file 1 — Additional file 1: Table S1. 91 DEGs were identified in GSE44031 series. [file 40001_2024_1643_MOESM1_ESM.pdf]

Table S1 91 DEGs were identified in GSE44031 series

**Series**	**DEGs**
GSE44031	Fam110a, Fuca1, Spata2, Strn3, Slit3, Nap1l5, Pold1, Efr3a, Cyp7b1, Bmp4, Prepl, Slc22a5, Dlst, Enox1, Cyb5r1, Lias, Hmgcl, Prpsap2, Znf622, Spag7, Zmiz1, Crmp1, Pgcp, Mpzl1, Acbd4, Fga, Slc1a4, Adfp, Sirpa, Hpca, C4b, RGD1563342, Eepd1, Lpar3, Pcp4l1, Ppap2b, Ccdc41, Tanc1, Lca5l, Ehbp1, MGC112715, Aldh9a1, Zc3h10, Reg1a, Psrc2, Cfh, Iqcf3, LOC501110, Stmn4, Cacnb1, Psme4, Inhba, Aif1, Mctp2, Hmgb3, Prpg1, Nmral1, Lgals9, LOC680231, Zfp238, Pla2g7, Cdc42bpb, Suds3, Nek3, Cant1, Rimklb, Ict1, Tpst1, Cdh3, Slc31a1, Taf1, Ankrd26, C2, Ppil1, Etaa1, A2m, Fkbpl, Klhl34, Usp48, Lap3, Siglec5, Scgb3a2, Ctr9, Rabl2b, Txnip, Wnt2, Gfap, Ptpro, Kit, Gadd45a, S100a4.
