# Supplementary material for: Identification of potential crucial genes and therapeutic targets for epilepsy
Source: Eur J Med Res. 2024 Jan 11;29:43. doi: 10.1186/s40001-024-01643-8 (PMC10782668; doi:10.1186/s40001-024-01643-8)
Supplement: Supplementary file 2 — Additional file 2: Table S2. 425 DEGs were identified in GSE1834 series. [file 40001_2024_1643_MOESM2_ESM.pdf]

Table S2 425 DEGs were identified in GSE1834 series

Series	DEGs
GSE1834	Insig1, Cd44, Hps3, Dnajb1, Pou3f1, Hnmt, Iapp, Hspa5, Cd74, Tgfb1, Dusp1, Pcmtd2, Fmo1, Mlst8, Nqo1, Mgst3, S100a10, Pla2g4a, Tap1, Flnc, Trh, Pacs1, Mybbp1a, Cth, Wdr37, Chn1, Camkv, Nell1, Ghr, C1qb, Ptger4, Ret, Mfhas1, Pdp1, Egfl7, Scn8a, Aprt, Asgr1, Pcna, Cfap97, Gas5, Adrb1, Egr1, Lcn2, Gabbr1, Dusp6, Angpt2, Prkar2b, Chrna4, Cited2, Plod3, Slc15a2, Txnrd1, Ung, Tspo, Alcam, Serp1, Dclk1, Gucy1a3, Myl12a, Slc6a17, Atxn3, Crem, Cebpd, Odc1, Postn, Crhbp, Arl11, Cyp4f1, Spon1, Ccnd1, Cnga3, Kif11, Pla2g5, Gss, Ly86, Decr2, Capg, Rnf112, Cyr61, Cald1, Bub1b, Mafb, Msn, Dcn, Cnksr3, Npepo, Ier3, Irf1, Emp1, Ddx39a, Ctsk, Tph1, Mfge8, Zfp36, Prkcd, Ddx21, Hmga1, Rnh1, Egr3, C1s, Plcd1, Atp1a2, Sdc1, Cyba, Hic2, Gpr12, Eif4e2, Stat1, Sstr1, Jak2, Gal, Hsph1, Junb, Scn2a, Prkab1, Arf6, RGD621098, Krt5, Anxa1, Ptgis, Gpt, Sdc4, Ripk3, Sgk1, Fn1, Mmp13, Obp3, Fosl1, Fkbp11, Igfbp3, Kbtbd3, Grifin, Rhoc, Klf10, Gabra5, Drd1, Pde4b, Nr3c2, Epb41l4b, Vav1, Ccl4, Hmox1, Nptx2, Itpr1, Syne1, Prpsap1, Spp1, Nfkbia, RGD1565616, Ocln, Myo5b, Ptbp1, Itpka, Ddit3, Aldoc, Nefl, Nr4a3, Rictor, Tpm4, Rbpj, Cntn6, Pnlip, Adm, LOC681290, Cyp2d4, Nes, Ssbp2, Rgs4, Cacna1d, Idi1, H19, Hbegf, Ece1, Anxa2, Tst, LOC102553736, Nln, Pde4d, Ppp,1r12a, Sult2a6, Adcy4, Klhl14, Rps27l, Ezr, Ambp, Rxrg, Ralbp1, Rab26, Ndufa4l2, Faim2, Gzmb, Smad2, Fcgr2b, Tnfrsf1a, Vom2r31, Mgp, Timp1, C3, Cbs, Myc, Ckmt1b, Abcb11, Serpine1, Camkk2, Gbp2, Lxn, Btg2, Thrsp, Crh, Igkc, Pdcl, Tlcd1, Aldh1a1, Pygm, Arc, Chka, Camk2a, Hspa2, Eif4ebp1, Myo,1e, Kras, Tac1, Neurod1, Kcnc3, Ptpn6, Ptgfr, Rab38, Vgf, Psmb8, Ddah1, Pdpn, Mdfic, Socs3, Nrn1, Irs2, Tars, Cd9, Plin2, Ibsp, Gclc, Tlr7, Tnfsf13, Adcyap1r1, Hk2, Cebpb, Meox2, Plcb1, Sult1d1, Litaf, Cd63, P4hb, Ptp4a1, Cadps2, Cd14, Scg2, Slco2a1, Irf7, Ptpn1, Nr1d2, Grin2d, Prkcdbp, Tpm3, Emd, Myh11, Sele, Nrg1, Cav1, Twf1, Slc5a3, Srd5a3, Acvr1c, Kcnd2, Il4r, Fosl2, Mcm5, Cyp2a3, Rhbdl1, Ccl2, Itga7, Serpinh1, L3hypdh, Lox, Klf4, Tnfsf4, Lin7b, Jun, Vim, Pkib, Plaur, Slc41a2, Plagl1, Hes5, Nr4a1, Tbc1d2b, Ctsc, Calca, Galr1, Ntf3, Syt4, Arrb1, Pla1a, Ogg1, Tjp2, Ptgs2, Lgals3, Pstpip1, Prb1, C1r, Cwc25, Cnn3, Fabp5, Aspg, Gria1, Zeb1, Pdyn, Egr2, Map1a, Nppc, Dbp, Dnajb5, Rbp1, Icam1, Slc6a1, Tnc, Ifitm3, Dync1i1, Gfra1, Slc8a1, Homer1, Slc3a2, Mat2a, Bdnf, Cd24, Hnrnpf, Pnoc, Hmgcr, Fos, Syt10, Mark2, Gch1, Degs2, Sox11, Cdv3, Pdlim4, Pim1, Gstt1, Cryab, Vsir, Casp6, Padi2, Nxt1, Bgn, Gdf10, Capn2, Rasl11b, Olr1, Rln1, Pmepa1, Atf3, Errfi1, Mapkapk3, Mcm6, Ms4a2, Edn2, Cbr1, Cxcl10, Grm3, Arl4a, Nae1, Tacr3, Fst, Ccl3, Dspp, Ppp,1r15a, Fam32a, Aldh1l1, Ncan, Lgals1, Arpc1b, Ifrd1, Dusp5, Ren, Gsta1, Ampd1, Orai1, Hmgcs2, Nr4a2, Hspa1a, RT1-Da, PVR, Plpp3, Tagln, C8b, Pam, Slc37a1, Spn, LOC102547700, Speg, Pxmp2, Rheb, Egr4, Mcf2l, Cxcl1, Sprr1a, Arpp21, Htr5b, Chrm1, Prkcg, Gabrg3, Agt, Adra1d, Ckmt2, Mvp, Penk, Gpc3, Serpina3n, Tmsbl1, Crispld2, Hspb1, Gfap, Ptpro, Kit, Gadd45a, S100a4.
