# Supplementary material for: Identification of potential crucial genes and therapeutic targets for epilepsy
Source: Eur J Med Res. 2024 Jan 11;29:43. doi: 10.1186/s40001-024-01643-8 (PMC10782668; doi:10.1186/s40001-024-01643-8)
Supplement: Supplementary file 3 — Additional file 3: Table S3. 5 overlapping DEGs were identified in GSE1834 and GSE44031. [file 40001_2024_1643_MOESM3_ESM.pdf]

Table S3 5 overlapping DEGs were identified in GSE1834 and GSE44031

**Series**	**Overlapping DEGs**
GSE1834, GSE44031	Gfap, Ptpro, Kit, Gadd45a, S100a4.
